# Radiation-induced microrna-622 causes radioresistance in colorectal cancer cells by down-regulating Rb

**DOI:** 10.18632/oncotarget.3762

**Published:** 2015-04-18

**Authors:** Wenhui Ma, Jiang Yu, Xiaolong Qi, Li Liang, Yan Zhang, Yi Ding, Xiaoshan Lin, Guoxin Li, Yanqing Ding

**Affiliations:** ^1^ Department of General Surgery, Nanfang Hospital, Southern Medical University, Guangzhou, China; ^2^ Department of Pathology, Nanfang Hospital, Southern Medical University, Guangzhou, China; ^3^ Department of Radiation Oncology, Massachusetts General Hospital and Harvard Medical School, Boston, MA, USA; ^4^ Department of Radiation Oncology, Nanfang Hospital, Southern Medical University, Guangzhou, China; ^5^ Department of Gastroenterology, Tongji Hospital, Tongji University School of Medicine, Shanghai, China

**Keywords:** radiosensitivity, microRNA-622, apoptosis, RB1, Rb

## Abstract

The standard treatment for patients with locally advanced rectal cancer is preoperative 5-fluorouracil-based chemoradiotherapy followed by total mesorectal excision. However, tumor response to standard dose radiation varies. In this study, we found that miR-622 was increased significantly in ionizing radiation-treated colorectal cancer (CRC) cells compared to the cells cultured with irradiated medium, and persisted stably in surviving cells treated with continuous low-dose radiation. Overexpression of miR-622 induced the radioresistance *in vitro*. In addition, miR-622 inhibited Rb expression by directly targeting RB1-3′UTR. Overexpression of Rb reversed miR-622-induced radioresistance *in vitro*. In response to ionizing radiation, the Rb-E2F1-P/CAF complex activated proapoptotic genes. Importantly, miR-622 was highly expressed in tumors of rectal cancer patients with non-regression after standard dose radiotherapy. In conclusion, miR-622 overexpressing cells are induced or selected by radiotherapy, causing in turn radioresistance and poor response to further therapy. MiR-622 is a potential biomarker of responders for radiotherapy and a potential therapeutic target.

## INTRODUCTION

Loco-regional recurrence after total mesorectal excision (TME), partially as a consequence of radioresistance, is difficult to treat and associated with poor survival for patients with colorectal cancer (CRC) [1]. Preoperative neoadjuvant chemoradiotherapy followed by TME has become the standard treatment for advanced, mid-low rectal cancer [2]. However, only approximately 20% of patients achieved pathological complete regression, whereas the rest showed an incomplete or no response [3]. Identification of biomarkers to predict survival outcome will promote the development of effective therapeutic strategies and eventually reduce the morbidity of patients with metastatic CRC.

MicroRNA (miRNA) is a small (18–25 nt), noncoding RNA molecule that represses protein translation through binding to the 3′-untranslated region (UTR) of their target mRNAs in a sequence-specific manner, causing mRNA degradation or suppressing mRNA translation [4, 5]. MiRNA expression varies from atypical hyperplasia, adenoma to carcinoma with the biological processes of CRC characterized by tissue specificity [6]. In recent years, miRNAs are well demonstrated to play a significant role in the mechanism of carcinogenesis and the prediction of therapeutic efficacy [7]. Previous studies also suggested the importance of miR-137, miR-224, miR-30 b, miR-26, miR-25 and miR-7 in viability, proliferation and invasion of tumor cells [8–13].

Recently, a specific signature profile of miRNA expression was detected in the pre-chemoradiotherapeutic clinical specimen of locally advanced rectal cancer [14]. The profile revealed the potential significance of several miRNAs as biomarkers with excellent sensitivity and specificity, however, the involved signaling pathway has not been elaborated [14]. Most recently, an individual signature of chemoradiosensitivity was established based on the genome-wide miRNA profiling in a CRC model *in vitro* [15].

MicroRNA-622 (miR-622) was identified as a CRC-related miRNA by miRNAs serial analysis of gene expression [16]. Functional analysis of miR-622 showed that it could suppress the growth of lung cancer and enhance the anticarcinogenic effect of resveratrol [17]. Furthermore, miR-622 is overexpressed in taxol-resistant ovarian cancer and is a significant prognostic marker for chemoresistant patients [18]. Despite the growing evidence highlighting the importance of miR-622 in carcinogenesis, none of studies investigated systematically its influence on radiotherapy in patients with CRC.

Retinoblastoma gene (RB1), as a member of pocket protein family with p107 and p130, is the first identified tumor suppressor [19]. Retinoblastoma protein (Rb) is involved in the process of DNA damage, repair, and replication, and plays a protective role against cell apoptosis and differentiation [20]. Although the majority of tumors happen to be deficient in or contain mutant RB1, wild-type (WT) RB1 is conserved in patients with CRC. Currently, the role of Rb in apoptosis in response to DNA damage or oncogenic stress is still controversial, which may be dependent on the phase of cell cycle [21–23].

In the study, we conducted a CRC-related miRNA screen in CRC cells treated with ionizing radiation (IR) aiming to identify the miRNAs associated with radiotherapy responses. The representative miRNA was then functionally analyzed for its potential influence on radio-resistance.

## RESULTS

### MiR-622 expression was increased in response to IR

**Figure 1:**
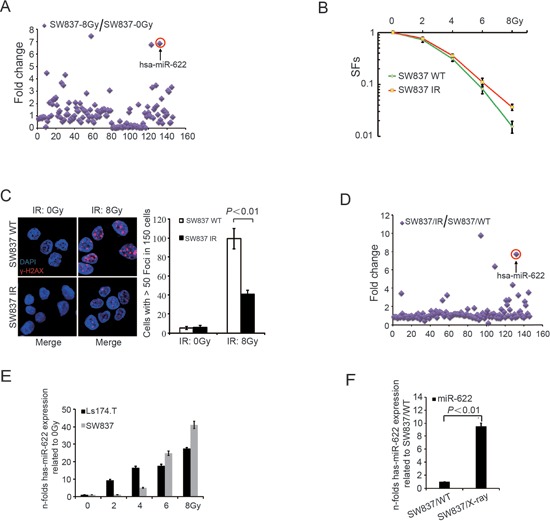
MiR-622 is induced by IR and maintained in surviving cells **A.** Quantitative analysis of tumor related miRNAs after exposed to X-Ray (8Gy, 1.5Gy/min). **B.** SFs was calculated as the number of colonies counted/(the number of cells seeded × plating efficiency/100). **C.** IF and quantitative analysis of γ-H2AX in surviving cells after IR (2Gy/day) for 7 days and wild-type SW837 cells treated with 8Gy. **D.** Quantitative analysis of tumor related miRNAs in surviving cells after IR (2Gy/day) for 7 days. **E.** Fold-change of miR-622 in response to different doses of IR. **F.** Fold-change of miR-622 in surviving cells after IR (2Gy/day) for 7 days compared to WT SW837 cells. RNU6B was an internal control of miR-622 by qRT-PCR. IR = ionizing radiation, SFs = survival fractions, IF = immunofluorescence.

To determine the sensitivity of five CRC cell lines to IR, we initially detected SFs of five cell lines by clonogenic survival assay. As shown in Supplementary Figure 1A, surviving fractions (SFs) of HT29 and SW480 cells were significantly higher than that of SW837, HCT116, and Ls174.T (*P* < 0.01; HT29 vs SW480, *P* = 0.05; HCT116 vs Ls174.T vs SW837, *P* = 0.24). Instantaneous DNA damage was analyzed to detect γ-H2AX expression at 24 hours after IR (Supplementary Figure 1B and 1C). HT29 and SW480 cells showed less γ-H2AX expression after irradiation at 8Gy compared with other cell lines. To identify the variated profiles of miRNAs after IR, SW837, an IR-sensitive rectal cancer cell line, was utilized for further experiments. As shown in Supplementary Figure 2A, cells were exposed to IR with 8Gy and tumor-related miRNAs were quantified. The expression of 26 miRNAs increased more than 2-fold in IR-treated cells compared to cells cultured with irradiated medium (Figure 1A). In order to investigate the sensitivity of CRC cells to IR and the change of miRNA expression levels after continuous low-dose radiation, SW837 was exposed to IR (2Gy/day) for 7 days (Supplementary Figure 2B). Despite mass mortality of cells, the surviving SW837 continuely proliferated and megascopic clones were observed after abolishment of irradiation for 7 days. Interestingly, higher SFs were observed in clones formed by IR-surviving cells compared to WT cells (*P* = 0.03, Figure 1B) and the number of cells with greater than 50 foci of γ-H2AX was significantly decreased in SW837/IR (*P* < 0.01, Figure 1C). Besides, miRNA expression profiles of the surviving cells suggested that the expression of 14 miRNAs increased more than 2-fold compared to WT cells (Figure 1D). Among them, 10 tumor associated miRNAs (miR-378a-3p, miR-424-5p, miR-206, miR-127-3p, miR-376c, miR-328, miR-217, miR-512-5p, miR-202-5p and miR-622) inreased significantly both in radiation-treated cells (8Gy for once) and surviving cells treated with continuous low dose irradiation (2 Gy/day for 7days). The functional relevance of miR-622 was further evaluated based on higher significance in both strategies. qRT-PCR analysis of miR-622 was performed in SW837 and Ls174.T exposed to increasing IR doses. As shown in Figure 1E, miR-622 expression increased dose-dependently in both cell lines (*P* < 0.01). Meantime, surviving cell clones after continuous low-dose radiation also charicterized with higher miR-622 exprssion (*P* < 0.01, Figure 1F).

### MiR-622 is associated with radiosensitivity of CRC cells *in vitro*

**Figure 2:**
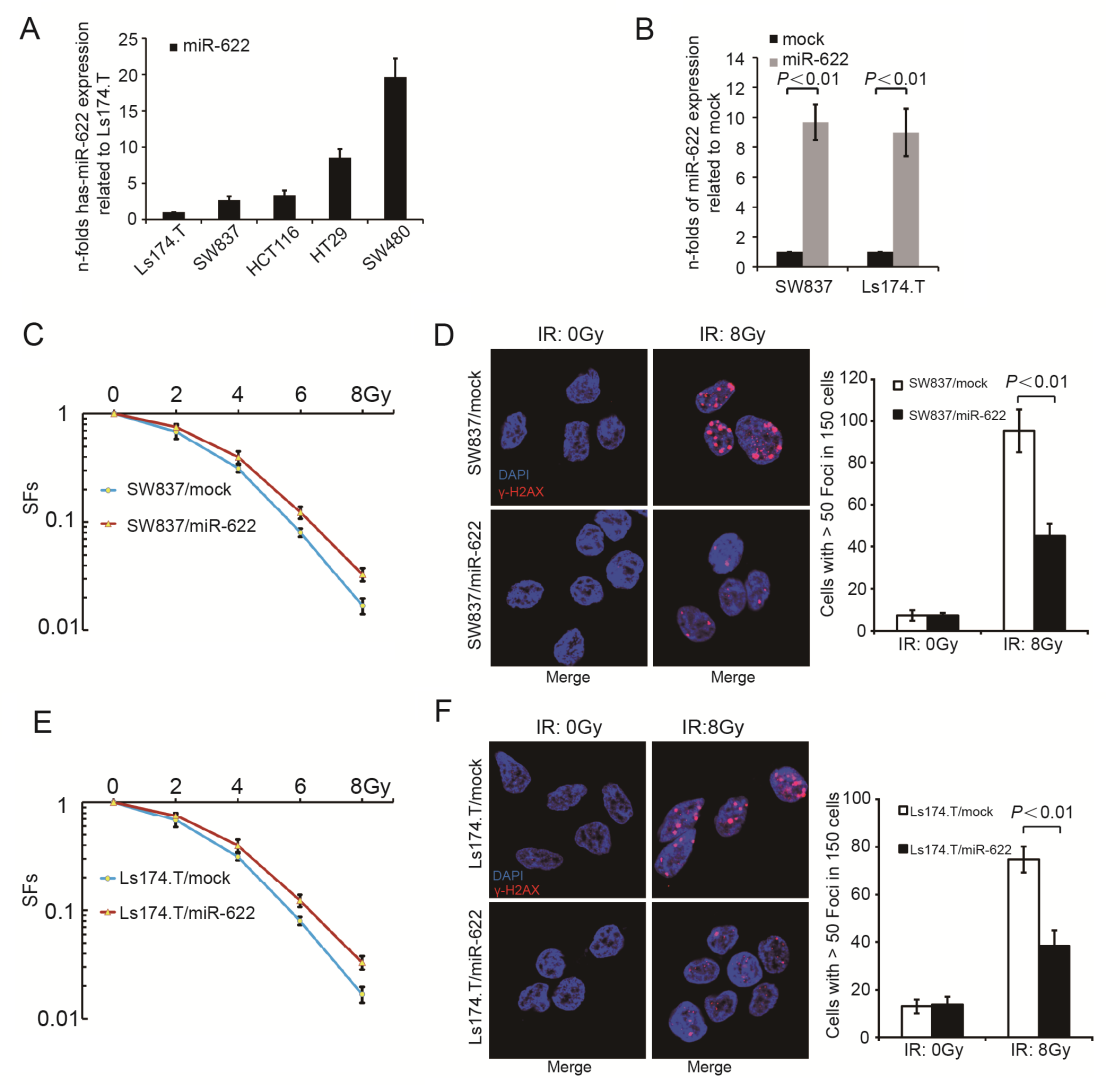
MiR-622 is associated with radiosensitivity of CRC cells *in vitro* **A.** Relative miR-622 expression of five CRC cell lines. **B.** Efficiency of transfection by lentivirus-miR-622 and lentivirus-miR-control. **C.** SFs of SW837 cells transfected by lentivirus-miR-622 and lentivirus-miR-control. **D.** IF and quantitative analysis of γ-H2AX in SW837 cells transfected by lentivirus-miR-622 and lentivirus-control. **E.** SFs of Ls174.T cells transfected by lentivirus-miR-622 and lentivirus-miR-control. **F.** IF and quantitative analysis of γ-H2AX in Ls174.T cells transfected by lentivirus-miR-622 and lentivirus-miR-control. RNU6B was an internal control miR-622 by qRT-PCR. CRC = colorectal cancer, SFs= survival fractions, IF = immunofluorescence.

The expression of miR-622 in five CRC cell lines showed a negative correlation with radiosensitivity (Figure 2A). Then, SW837 and Ls174.T were transfected with miR-622 expressing and miR-control lentivirus, respectivrly. The efficiency of transfection was as following: SW837/mock vs SW837/miR-622, *P* < 0.01; Ls174.T/mock vs Ls174.T/miR-622, *P* < 0.01) (Figure 2B). In addition, overexpression of miR-622 caused a significant increase of SFs in both cell lines compared to miR-control lentivirus transfected ones (Figure 2C, *P* < 0.01; Figure 2E, *P* = 0.04). Meanwhile, γ-H2AX foci in X-Ray exposed SW837/miR-622 and Ls174.T/miR-622 cells was significantly decreased compared to miR-control lentivirus transfected ones (Figure 2D, *P* < 0.01; Figure 2F, *P* < 0.01). Therefore, miR-622 inhibited the response of CRC cells to IR *in vitro*. Furthermore, we knocked down the expression of miR-622 in SW480 and HT29 cell lines by transfecting anti-miR-622 inhibitors. The results showed that down-regulation of miR-622 caused a significant decrease of SFs in both cell lines (Supplementary Figure 3A, *P* < 0.01 and Supplementary Figure 3C, *P* < 0.01). In addition, the expression of γ-H2AX was much higher in SW480 and HT29 cells transfected with anti-miR-622 inhibitors compared to those transfected with inhibitor control (Supplementary Figure 3B, *P* < 0.01 and Supplementary Figure 3D, *P* < 0.01).

### MiR-622 targets directly the tumor suppressor RB1

**Figure 3:**
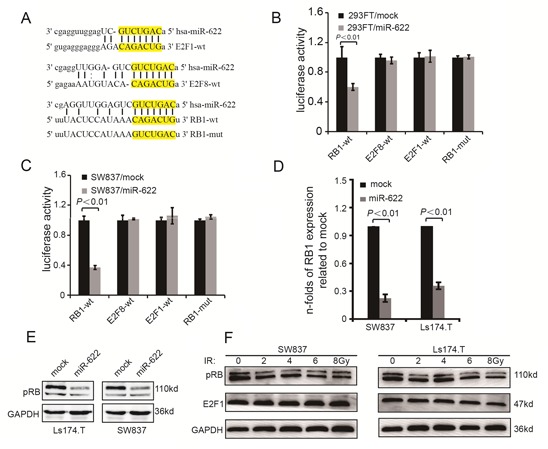
MiR-622 targets directly the RB1 **A.** Schematic illustration of the potential miR-622-binding sites in 3′-UTRs of E2F8, E2F1, and RB1. **B.** Luciferase activity of 293FT cells transfected with luciferase vectors containing wild-type or mutant plasmids and mimics-NC (0.5 uM) or mimics-miR-622 (0.5 uM), respectively. **C.** Luciferase assay of SW837 cells transfected with the indicated reporters and mimics-miR-622 or mimics-control. **D.** Folds change of RB1 in mRNA level in cells transfected with lentivirus-miR-622 and lentivirus-miR-control. **E.** Rb expression variation of cells transfected by lentivirus-miR-622 and lentivirus-miR-control analyzed by Western blotting. **F.** Rb and E2F1 variation in response to radiation. RNU6B and GAPDH were internal controls of miR-622 and RB1 for qRT-PCR. GAPDH was an internal control of Western blotting.

An exhausitive search based on three bioinformatics algorithms (microRNA.org, miRDB, TargetScan) was performed to predict miR-622 targeted mRNAs. Three theoretical target genes of miR-622 (E2F1, E2F8, and RB1, Figure 3A) related to apoptosis or cell cycle were confirmed using luciferase assay. 3′UTR fragments of mRNAs candidates containing the WT miR-622-binding site were subcloned and inserted into a luciferase reporter plasmid. Transient transfection of WT luciferase reporter plasmid with miR-622-mimics into 293FT and SW837 cells led to a visible decrease in luciferase activity of the plasmid containing RB1 3′UTR (Figure 3B, *P* < 0.01 and Figure 3C, *P* < 0.01). When miR-622-binding sites in the 3′UTR of RB1 mRNA were mutated, luciferase activity of RB1-mut was restored.

To clarify the relationship between endogenous miR-622 and RB1 expression, the concentration of Rb encoded by RB1 were analyzed in five CRC cell lines. In contrast to miR-622 expression, the level of Rb expression in SW837, Ls174.T, and HT116 were higher than that in HT29 and SW480 (Supplementary Figure 3). Furthermore, overexpression of miR-622 significantly decreased the expression of RB1 and Rb in SW480 and Ls174.T (Figure 3D and Figure 3E). Meanwhile, when SW837 and Ls174.T cells were exposed to increasing doses of radiation, the expression of Rb decreased correspondingly (Figure 3F). However, the level of E2F1, a protein closely connected with RB1, did not change in response to increasing IR. Therefore, miR-622 inhibited Rb by directly targeting the 3′UTR of RB1 mRNA.

### Overexpression of Rb reverses radioresistance induced by miR-622 *in vitro*

**Figure 4:**
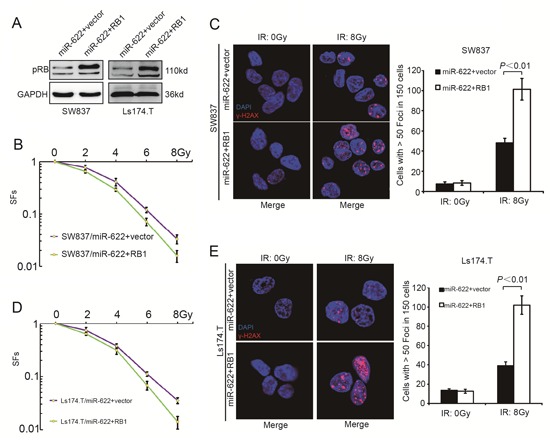
RB1 reverses miR-622 induced radioresistance *in vitro* **A.** Rb expression variation of miR-622 overexpressed cells transfected by lentivirus-RB1 and lentivirus-vector control analyzed by Western blotting. **B.** SFs of the miR-622 overexpressed SW837 cells transfected with lentivirus-RB1 and lentivirus-vector control. **C.** IF and quantitative analysis of γ-H2AX in miR-622 overexpressed SW837 cells transfected with lentivirus-RB1 and lentivirus-vector control. **D.** SFs of the indicated cells transfected with lentivirus-RB1 and lentivirus-vector control. **E.** IF and quantitative analysis of γ-H2AX in the indicated cells. GAPDH was an internal control of Western blotting. SFs = survival fractions.

A rescue experiment was further performed to explore whether Rb could functionally reverse the radioresistance induced by miR-622. pPRIME-GFP-RB1 without 3′UTR and pPRIME-GFP-control vector were transfected into lentiviral packaging cell lines 293T. Lentivirus containing RB1-expressing and control vector were then used to transfect SW837/miR-622 and Ls174.T/miR-622 cells. As a result, both cell lines showed an increase of Rb level (Figure 4A). When cells were treated with increaing doses of IR, the SFs decreased remarkably after overexpression of Rb compared to those transfected with vector control lentivirus (Figure 4B, *P* < 0.01 and Figure 4D, *P* < 0.01). Meanwhile, Rb overexpression resulted in a relatively higher level of γ-H2AX in both SW837/miR-622 and Ls174.T/miR-622 cells (Figure 4C, *P* < 0.01 and Figure 4E, *P* < 0.01). The rescue experiment indicated that Rb could reverse the radioresistance induced by miR-622 *in vitro*.

### Proapoptotic genes are activated transcriptionally by Rb-E2F1-P/CAF complex in response to radiation

**Figure 5:**
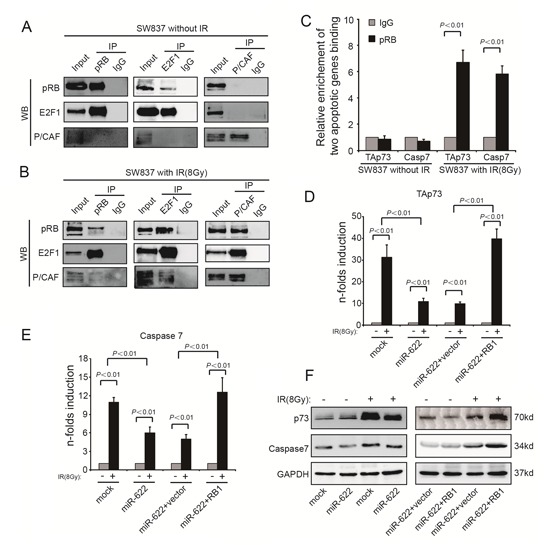
TAp73 and Caspase7 are activated by Rb-E2F1-P/CAF complex **A.** and **B.** Relationship among Rb, E2F1, and P/CAF before and after IR analyzed by co-immunoprecipitation followed by Western blotting. **C.** Chromatin immunoprecipitation analysis of Rb binding to TAp73 and Caspase7 with/without IR. **D.** and **E.** TAp73 and Caspase7 expression in the indicated cells with/without IR analyzed by qRT-PCR. **F.** p73 and Caspase7 expression in the indicated cells with/without IR analyzed by Western blotting. GAPDH was an internal control for both qRT-PCR and Western blotting. IR = ionizing radiation.

DNA damage triggers the histone acetyltransferase P/CAF to acetylate E2F1. This modification favors the formation of transcriptionally active Rb-E2F1-P/CAF complex. Therefore, we assessed the association of Rb, E2F1 and P/CAF in SW837 before and after radiation. For unexposed SW837 cells, P/CAF was not associated with either Rb or E2F1 (Figure 5A), whereas it was recruited to Rb and E2F1 after IR (8Gy) (Figure 5B). Furthermore, Rb, regulated by E2F1 in response to IR, was also comfirmed to participate in the transcription of proapoptotic genes TAp73 and Caspase7 by chromatin immunoprecipitation assay (Figure 5C). According to the results of qRT-PCR, overexpression of miR-622 depressed the expression of TAp73 and Caspase7 in response to IR, however, overexpression of Rb significantly promoted IR-induced proapoptotic genes (Figure 5D and Figure 5E). Besides, ectopic expression of miR-622 also supressed TAp73 and Caspase7 in protein level (Figure 5F). Therefore, miR-622 and Rb showed a significant regulatory role in the transcription of proapoptotic genes.

### MiR-622 shows a high expression in rectal cancer patients with non-regression after-radiation therapy

**Figure 6:**
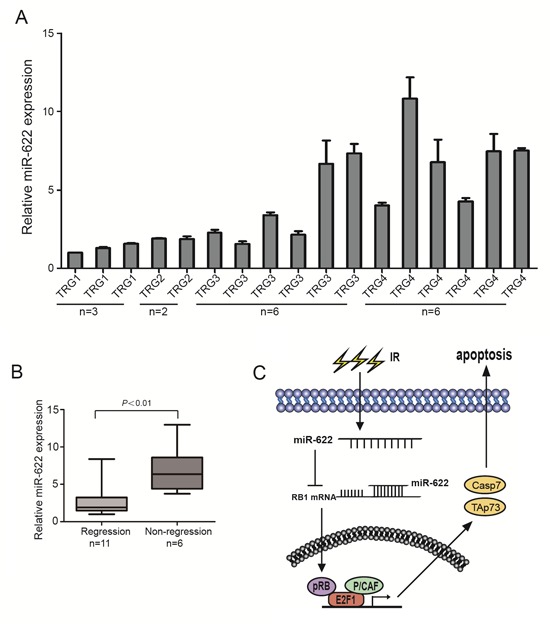
MiR-622 shows a high expression in rectal cancer patients with non-regression after standard dose radiation therapy **A.** Relative expression of miR-622 in biopsies of 17 rectal cancer patients before any treatment. **B.** Analysis of miR-622 expression between patients without regression (TRG4) and partial/complete regression (TRG1-3). **C.** The role of miR-622 in radioresistance of colorectal cancer. RNU6B was an internal control for qRT-PCR. TRG = tumor regression grade.

17 participants with T3-4/N+ rectal cancer received standard dose radiation therapy followed by TME surgery were enrolled in the study. Their frozen biopsies before treatment were obtained and miR-622 expression was analyzed by qRT-PCR. Pathologic response was scored according to the Mandard TRG scale by two independent pathologists. As a result, 6 patients characterized with non-tumor regression (TRG4) exhibited higher expression of miR-622 (Figure 6A). In addition, a significant difference of miR-622 expression between patients without regression (TRG4) and partial/complete regression (TRG1-3) was verified by Whiskers diagrams analysis (Figure 6B). These results indicated the potential of miR-622 as a biomarker for rectal cancer patients to predict their response to radiation treatment.

## DISCUSSION

Non-pathologic regression of tumor after standard dose radiation occurs in about 20% of rectal cancer and is always correlated with a poor prognosis [24, 25]. An efficient biomarker to predict pathologic responses and the involved mechanism of radioresistence is urgently needed. The ultimate goal is to select more effective and less toxic treatment for each patient. In this study, we assessed the radiosensitivity of five CRC cell lines and analyzed varied miRNA expression of SW837 in response to IR that showed higher sensitivity. This is the first study to detect the miRNA profile in CRC cells after two radiation strategies. According to the results, miR-622 increased obviously after receiving IR and maintained at a high level in survival cells. Our data suggested that the ectopic expression of miR-622 dramatically blocked γ-H2AX and promoted the survival of CRC cells in response to IR *in vitro*.

Parts of identified miRNAs in the study have been reported [14, 15]. For instance, miR-224 was associated with decreased cell viability, increased sensitivity to apoptosis and increased resistance to methotrexate therapy in CRC [26]. MiR-326 was positively correlated with doxorubicin sensitivity through down-regulation of multidrug resistance-associated proteins in breast cancer [27]. These results suggested that miRNAs could affect the sensitivity through repressing their target genes. We, therefore, aimed to functionally validate the role of miR-622 in radioresistence of CRC. Our data showed that miR-622 integrated to the complementary sites of the 3′UTR of RB1, and dramatically decreased the expression ofRb. In addition, a visible inverse correlation between miR-622 and Rb was observed in CRC cell lines. These results provided the solid evidence that miR-622 could act in the regulation of RB1. Besides, we raised the concept for the first time that Rb could participate in transcriptionally active complexes. The analysis of RB1 mutant embryos resulted in the prevailing view that Rb had an anti-apoptotic function [28]. However, it is now clear that much of this effect results from a defect of proliferation in extraembryonic tissues rather than cell autonomously [29, 30]. Loss of Rb did promote apoptosis in a cell-autonomous manner in some tissues of the developing embryo, and this appeared to reflect an inability to undergo terminal differentiation [31]. Moreover, some cell types, such as mouse embryonic fibroblasts, had a heightened sensitivity to DNA damage in the absence of Rb [32, 33]. However, this anti-apoptotic function of Rb is not always observed. Both pro-apoptotic and anti-apoptotic functions of Rb had been reported before [21, 23]. Rb was shown to cooperate with differentiation-specific transcription factors in the activation of key target genes. The formation of Rb-E2F1-P/CAF complex promoted the apoptosis of proliferating cells in response to DNA damage [23]. IR treatment led to the formation of a transcriptionally active E2F1-Rb-P/CAF complex on multiple proapoptotic gene promoters, thereby activating their transcription. In the study, we demonstrated that overexpression of miR-622, which inhibited Rb, was merely permissive for the formation of Rb-E2F1-P/CAF complex and reduced the expression of proapoptotic genes in response to radiation, which led to the resistance of CRC to radiotherapy. However, overexpression of Rb could effectively rescue miR-622-induced radioresistance *in vitro*. Finally, rectal cancer patients with overexpression of miR-622 exhibited non-response to standard dose radiation therapy. Therefore, we suggested miR-622 as a potential biomarker to identify responders for radiotherapy.

On the basis of our results, we propose a model that highlights the role of miR-622 in radiosensitivity of CRC. In this model, the up-regulation of miR-622 blocks its direct target gene, RB1, in CRC cells. Loss of Rb inhibits the formation of Rb-E2F1-P/CAF complex, which results in a decrease of proapoptotic genes TAp73 and Caspase7, and leads to the radioresistance (Figure 6C). However, it is noted that miR-622 could also be induced by radiation. Intriguingly, this is not without precedent. The concept of “selection for oncogenic resistance” was raised to explain why therapeutic response may not prolong the life of a cancer patient [34, 35]. The resistance mechanisms were divided into oncogenic (e.g. apoptosis avoidance, cell cycle dysregulation) and non-oncogenic (e.g. drug transporters, target mutation) [34, 35]. In our case, radiation treatment selected for expression of oncogenic alterations, such as miR-622, which conversely made cells adaptation, more oncogenic and resistant to radiotherapy. Those clones with radioresistance were eventually selected. However, the effect of miR-622 on CRC progression in addition to radioresistance needs to be further investigated.

In conclusion, we demonstrated the effect of miR-622 on radiosensitivity and its downstream mechanism for CRC *in vitro*. MiR-622 is proposed as a potential biomarker to identify responders for radiotherapy and an efficient therapeutic target for patients with CRC.

## MATERIALS AND METHODS

### Cell lines culture and transfection

Five CRC cell lines (SW480, HCT116, HT29, SW837, and Ls174.T) were cultured in RPMI 1640 (HyClone) supplemented with 10% heat-inactivated FBS (HyClone). Cells were purchased from the American Type Culture Collection and maintained at the Department of Pathology, Southern Medical University. The miR-622 mimics, mimics control, anti-miR-622 inhibitors and miR-inibitor control were purchased from GenePharma (GenePharma, Shanghai, China) and transfected instantaneous into CRC cells using Lipofectamine 2000 reagent (Invitrogen, Foster, USA). MiR-622 overexpression and miR-control lentivirus were purchased from Genechem (GENECHEM, Shanghai, China) for stable transfectants.

### Ionizing radiation and miRNA screen

The SW837 cells were plated in 100-mm dishes containing medium and 10% FBS for 24 h before IR and exposed to X-ray unit at a dose rate of 1.55 Gy/min. WT cells for control were cultured with previously irradiated medium and 10% FBS. Meantime, SW837 cells were exposed to IR (2Gy/day) for 7 days and the surviving cells were continue to culture until there was enough cells for analysis. miRNA qPCR arrays for profiling the expressions of CRC-related miRNAs and specific primers were designed by GeneCopoeia and added prior to 96 well plate (GeneCopoeia, Rockville, USA). Poly A Polymerase, RTase Mix, qPCR Mix, ROX Reference Dye, Universal Adaptor PCR Primer were purchased from GeneCopoeia (GeneCopoeia, Rockville, USA). RNU48 and U6 were detected and used as internal standards.

### Clinical specimen

The study was approved by the Ethics Committees of Nanfang Hospital, Southern Medical University. All participants gave written informed consent. Tumor tissues of 17 participants with a histologic diagnosis of rectal adenocarcinoma invading through the intestinal wall and/or with lymph node involvement as evaluated by endorectal ultrasonography/CT were collected before receiving neoadjuvent therapy (capecitabine + oxaliplatin in combination with 45Gy of pelvic conformal radiotherapy). Pathologic response was scored according to tumor regression grade (TRG) after 6 to 8 weeks of radiotherapy completion [36]. All participants then underwent surgery.

### Clonogenic survival assay

Cell lines were plated at specific cell densities, corresponding with the IR dose, in 60-mm dishes containing medium and 10% FBS (400 cells for 0 and 2 Gy, 800 cells for 4 Gy, 2000 cells for 6 Gy, 4000 cells for 8 Gy). IR and calculation procedure were implemented as previously described [37]. Cells exponentially growing were harvested by exposure to trypsin and counted. They were diluted serially to appropriate densities, and plated in dishes with complete medium. Cells in culture were exposed to 2, 4, 6, or 8 Gy radiation (X-rays using a cesium-137 source, 1.55 Gy/min). After 14 to 20 days of incubation, cells were stained with 0.5% crystal violet in absolute ethanol, and colonies with more than 50 cells were counted under a dissection microscope. Plating efficiency was defined as the percentage of cells seeded that grew into colonies under a specific culture condition. SFs, as a function of irradiation, was calculated as the number of colonies counted/(the number of cells seeded × plating efficiency/100). The SFs after indicated IR doses was used to determine radiosensitivity.

### RNA extraction and real-time quantitative PCR

Total RNA was extracted with TRIzol reagent (TaKaRa, Daian China). The All-in-oneTMmiRNA real-time quantitative PCR (qRT-PCR) Detection Kit (GeneCopoeia, Rockville, USA) was used for cDNA synthesis and quantitative detection of mature miRNAs with All-in-oneTMmiRNA qPCR Primers (GeneCopoeia, Rockville, USA). The primers were shown in Supplementary Table 1.

### Immunofluorescence for γH2AX

Immunofluorescence (IF) staining was conducted to detect the expression of γH2AX in cell lines exposed to different doses of radiation [38]. The number of cells with greater than 50 foci was calculated after cells were irradiated to 8 Gy for 24 hours. All precedures were carried out in triplicate.

### Plasmids construction and luciferase assay

Regions containing the miR-622-binding site of the human E2F1 3′UTR, E2F8 3′UTR and RB1 3′UTR were generated by PCR amplification and subcloned into the SacI/XmaI sites of the pGL3-basic luciferase reporter plasmid (Promega, Madison, USA). The pGL3-luciferase reporter gene plasmids (pGL3.0-E2F1-3′-UTR, pGL3-E2F8-3′-UTR, and pGL3-RB1-3′-UTR) or control-luciferase plasmid were cotransfected into cells with the control pRL-TK Renilla plasmid (Promega, Madison, USA) using Lipofectamine 2000 Reagent (Invitrogen, Foster, USA). Luciferase and renilla activities were assayed by the Dual Luciferase Reporter Assay Kit (Promega, Madison, USA) after transfection for 48 hours. All experiments were conducted three times and the data were presented as mean ± SD.

### Chromatin immunoprecipitation assays

According to the chromatin immunoprecipitation assay kit (Active motif, Carlsbad, USA), SW837 cells with or without IR were lysed using sodium dodecyl sulfate lysis buffer, and DNA was sheared by sonication to lengths between 200 and 1,000 base pairs. Protein-DNA complexes were precipitated by either anti-Rb antibody (Abcam, Boston, USA) or control IgG, followed by elution of the complex. Crosslinks in the protein-DNA complexes were then reversed by addition of NaCl. The primers were shown in Supplementary Table 1.

### Co-immunoprecipitation assays

For immunoprecipitation, cells were lysed using RIPA buffer (10 mM Tris-HCl, pH 7.4, 150 mM NaCl, 600 mM NP-40, 1% Triton X-100, 10% glycerol, 1 mM phenylmethylsulfonyl fluoride, 1mM sodium fluoride, and 1 mM sodium orthovanadate), and incubated with 20 ml protein-A Sepharose beads (Santa Cruz Biotechnology, Dallas, USA), 1mg anti-Rb (Abcam, Boston, USA) and IgG antibodies at 4°C overnight. The samples were then washed five times with PBS and prepared for Western blotting.

### Western blotting assays

Proteins were separated by electrophoresis based on its molecular weight and transferred from the gel to a PVDF membrane. Then, primary antibodies probing the target protein after the PVDF membrane were blocked by bovine serum albumin. The first antibodies were then detected by second antibodies, which could recognize them conjugated to enzyme horseradish peroxidase. The information of antibodies were as follows: anti-Rb (Epitomics, Burlingame, USA), anti-E2F1(Epitomics, Burlingame, USA), anti-P/CAF monoclonal antibodies (Santa Cruz Biotechnology, Dallas, USA), anti-p73 (Abcam, Boston, USA) and anti-Caspase7 (Abcam, Boston, USA).

### Statistical analysis

All statistical analyses were conducted by SPSS13.0. The two-tailed paired Student's *t* test was used to analyze two groups. The Mann-Whitney U test and Spearman's correlation analyses were utilized to analyze the relationship between miR-622 and clinicopathologic features of CRC. In addition, the clonogenic survival assay was conducted using one-way analysis of variance for factorial design. *P* < 0.05 was considered statistically significant.

## SUPPLEMENTARY FIGURES AND TABLE



## Abbreviations

CRC: colorectal cancer
IF: immunofluorescence
IR: ionizing radiation
MicroRNA: miRNA
Rb: retinoblastoma protein
qRT-PCR: real-time quantitative PCR
RB1: retinoblastoma gene
SFs: surviving fractions
TME: total mesorectal excision
TRG: tumor regression grade
UTR: untranslated region
WT: wild-type
