# Supplementary material for: Radiation-induced microrna-622 causes radioresistance in colorectal cancer cells by down-regulating Rb
Source: Oncotarget. 2015 Apr 18;6(18):15984–94. doi: 10.18632/oncotarget.3762 (PMC4599251; doi:10.18632/oncotarget.3762)
Supplement: Supplementary file 1 [file oncotarget-06-15984-s001.pdf]

SUPPLEMENTARY FIGURES AND TABLE

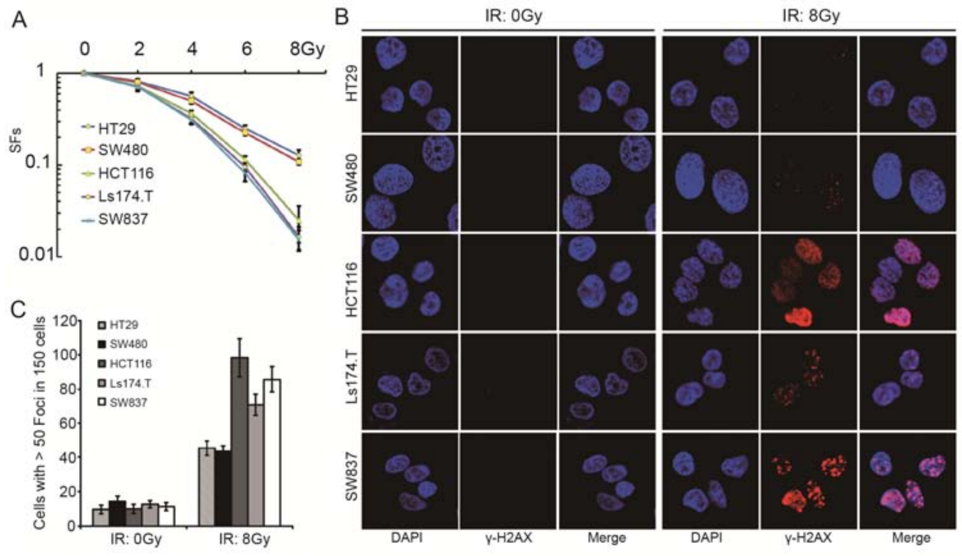

Supplementary Figure S1: SFs and γ-H2AX expression of five CRC cell lines in response to IR. A. SFs of five CRC cell lines were irradiated with graded doses of X-rays. SFs was calculated as the number of colonies counted/(the number of cells seeded × plating efficiency/100). **B.** and **C.** IF and quantitative analysis of γ-H2AX in five CRC cells. SFs = survival fractions, CRC = colorectal cancer, IF = immunofluorescence.

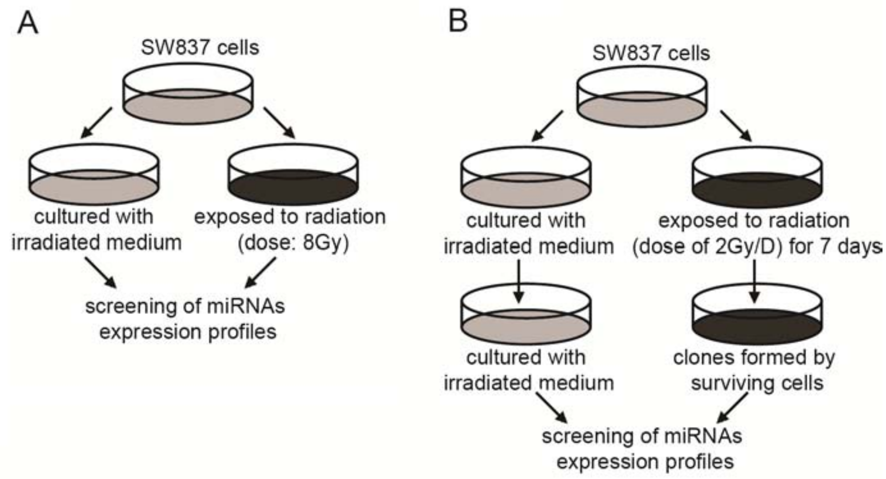

Supplementary Figure S2: Screening of miRNAs expression profiles. A. Schematic outline of tumor-related miRNA expression profiles in response to IR of SW837 cells. **B.** Schematic outline of tumor-related miRNA expression profiles in surviving SW837 cells after IR (2 Gy/day) for 7 days. IR = ionizing radiation.

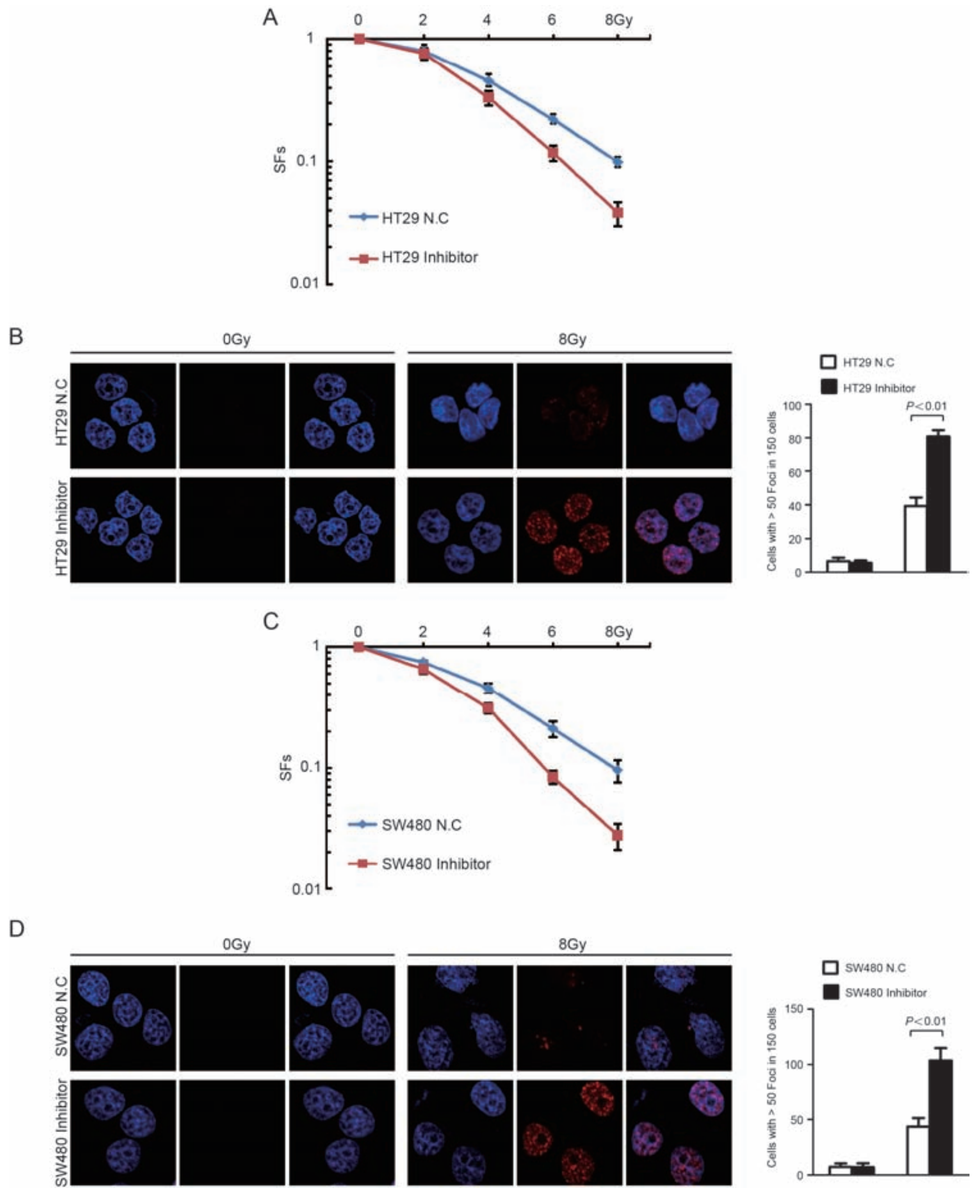

Supplementary Figure S3: Down-regulation of miRNA-622 promotes radiosensitivity of CRC in vitro. A. SFs of HT29 cells transfected by anti-miR-622 inhibitor and inhibitor control. **B.** IF and quantitative analysis of γ-H2AX in HT29 cells transfected by anti-miR-622 inhibitor and inhibitor control. **C.** SFs of SW480 transfected by anti-miR-622 inhibitors and inhibitor control. **D.** IF and quantitative analysis of γ-H2AX in SW480 cells transfected by anti-miR-622 inhibitors and inhibitor control. SFs = survival fractions, CRC = colorectal cancer, IF = immunofluorescence.

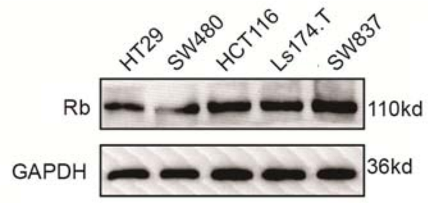

Supplementary Figure S4: Rb expression of five CRC cell lines. GAPDH was an internal control. CRC = colorectal cancer.

Supplementary Table S1. Sequence of Primer for ChIP, qRT-PCR and 3′UTR PCR

Gene	Sequence
**ChIP Primer**	
hCasp7 forward	5′-TTTGGGCACTTGGAGCGCG-3′
hCasp7 reverse	5′-AAGAGCCCAAAGCGACCCGT 3′
hTAp73 forward	5′-TGAGCCATGAAGATGTGCGAG-3′
hTAp73 reverse	5′-GCTGCTTATGGTCTGATGCTTATGG-3′
**qRT-PCR Primer**	
GAPDH forward	5′-CCATCAATGACCCCTTCATTGACC-3′
GAPDH reverse	5′-GAAGGCCATGCCAGTGAGCTTCC-3′
TAp73 forward	5′-CATGGAGACGAGGACACGTA-3′
TAp73 reverse	5′-CTGTAACCCTTGGGAGGTGA-3′
Caspase7 forward	5′-GCAGTGGGATTTGTGCTTCT-3′
Caspase7 reverse	5′-CCCTAAAGTGGGCTGTCAAA-3′
RB1 forward	5′-CTCTCGTCAGGCTTGAGTTTG-3′
RB1 reverse	5′-GACATCTCATCTAGGTCAACTGC-3′
**3′UTR PCR Primer**	
E2F1 forward	5′-ATTTCTGACAGGGCTTGGAGGGACC-3′
E2F1 reverse	5′-GGTTTTAATAAATATTTTGATGACGTT-3′
E2F8 forward	5′-TCAACAGATGTTGGCTTAGTTTAA-3′
E2F8 reverse	5′-CAAATTAAAACTTTTCTAAAATATA-3′
RB1 forward	5′-TTGTCTCTCACAGATGTGACTGTAT-3′
RB1 reverse	5′-AGGTCAAGGGCTTACTATTTCTGGG-3′
